# The Duration of Uncertain Times: Audiovisual Information about Intervals Is Integrated in a Statistically Optimal Fashion

**DOI:** 10.1371/journal.pone.0089339

**Published:** 2014-03-04

**Authors:** Jess Hartcher-O'Brien, Massimiliano Di Luca, Marc O. Ernst

**Affiliations:** 1 Multisensory Perception and Action Group, Max Planck Institute for Biological Cybernetics, Tübingen, Germany; 2 Cognitive Neuroscience Department and Cognitive Interaction Technology-Center of Excellence, Bielefeld University, Bielefeld, Germany; 3 Center for Computational Neuroscience and Cognitive Robotics and School of Psychology, University of Birmingham, Edgbaston, Birmingham, United Kingdom; 4 Bernstein Center for Computational Neuroscience, Tübingen, Germany; University of California, Merced, United States of America

## Abstract

Often multisensory information is integrated in a statistically optimal fashion where each sensory source is weighted according to its precision. This integration scheme is statistically optimal because it theoretically results in unbiased perceptual estimates with the highest precision possible. There is a current lack of consensus about how the nervous system processes multiple sensory cues to elapsed time. In order to shed light upon this, we adopt a computational approach to pinpoint the integration strategy underlying duration estimation of audio/visual stimuli. One of the assumptions of our computational approach is that the multisensory signals redundantly specify the same stimulus property. Our results clearly show that despite claims to the contrary, perceived duration is the result of an optimal weighting process, similar to that adopted for estimates of space. That is, participants weight the audio and visual information to arrive at the most precise, single duration estimate possible. The work also disentangles how different integration strategies – i.e. considering the time of onset/offset of signals - might alter the final estimate. As such we provide the first concrete evidence of an optimal integration strategy in human duration estimates.

## Introduction

Imagine you are attending a cellist concert. As the cellist drags the bow across the strings you try to guess how long that resonant note lasted by using two sources of sensory information: the duration of the sound and that of the bow movement. From these two partially conflicting sources of information (i.e. because of residual arm movements and room acoustics), your brain is attempting to obtain one unique estimate of duration. Duration can help structure, among other things, the rhythm of the music. From simple characteristics such as the duration of a musical note, to complex behaviours of anticipation, duration estimates of intervals in the millisecond-to-second range guide our perception of, and interactions with the environment (e.g., [1] [2]). Yet, the mechanisms accomplishing estimates of duration remain a contentious issue ([3]; [4]; [5]). Most information about the external world provides multiple sensory signals to your nervous system. These signals can be used independently to estimate properties of the environment – as such they are redundant. The present study addresses the question of how redundant auditory and visual cues specifying interval duration are integrated into a unique audiovisual estimate.

The integrated estimate of a redundantly specified property e.g., location or size, is known to be the result of a weighted average of the individual component estimates
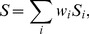
(1)where the weights are proportional to reliability of the estimates according to: 
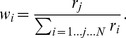
(2)
[6] where the reliability *r* is inverse variance of the estimates. With such weights, the integrated estimate has the highest possible reliability *r* and the integration is said to be “statistically optimal” - but with an extreme weight assigned to one sensory component there will be minimal benefit from integration. The model assumes that the estimates are unbiased with normally distributed noise that is statistically independent across estimates. This is the Maximum Likelihood Estimate (MLE) model [7]:
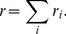
(3)Previous research suggests that estimates of elapsed time do not obey this integration strategy (e.g., [8]; [9]). Why should duration information be different from all the other cases where optimal integration has been observed? Duration estimates cannot be made while the information remains sensorially available as duration is defined only once the event has ended, and thus sensory information is no longer available. As such, duration estimates are post-hoc [10]. It has been hypothesized that this might be the reason why multisensory duration estimation would be suboptimal ([8]; [9]). The question is whether these suboptimal findings are specific to these studies or whether they highlight a general feature of duration estimation.

In previous studies (i.e. [8]; [11]), temporal intervals were defined by the time elapsed between two short stimuli (defined as markers) which could be auditory, visual, or audiovisual. The signal was only present at interval onset and offset not during the judged interval itself. Such a stimulus can be defined as an “empty interval” ([12]; [13]). This stimulus type is ambiguous regarding which temporal property is redundantly specified (i.e., *S* in Equation 1): the time points defined by the onset and offset markers or the duration in between those markers. It is therefore unclear which property undergoes multisensory integration. That is, one strategy to process the information is for participants to estimate duration of unisensory intervals separately and subsequently integrate them into a unified percept (we define this case as “redundant duration”). Alternatively, they could first combine audio and visual onset and offset markers, respectively and then estimate the duration between these two integrated time points (this is the “redundant time points” case). The mismatch between previous studies and an optimal strategy, might be due to this ambiguity: The MLE predictions from previous research have been based on the assumption that visual and auditory duration estimates are integrated, however, the empirical integration results could have been derived from estimates of the integrated markers. Using filled intervals, we show, for the first time that duration estimation follows an optimal integration rule and therefore we suggest that previously reported suboptimal behaviour may be the result of different mechanisms underlying the problem of obtaining duration from empty intervals, not the strategy used by the nervous system per se.

## Methods

### 2.1 Participants

Eight volunteers, all reporting normal hearing and normal or corrected-to-normal vision took part in the entire experiment. All participants were naïve to the purposes of the study. Participants gave their written, informed consent prior to the experiment and were naive to the purpose of the study. They received €8 per hour for their participation. The Ethics committee of the University of Tübingen gave approval for the study and for the consent form used to obtain written consent. The study was conducted in accordance with the Ethical guidelines expressed in the Declaration of Helsinki. Eleven participants began the experiment. Three participants' were excluded due to their inability to perform the uni-sensory discrimination task above chance (average Weber Fractions across all conditions exceed 3*σ from the mean Weber Fraction of all participants i.e. chance performance). If participants cannot perform in the uni-sensory task investigating integration is not useful. This left us with 8 participants who conducted the complete experiment.

### 2.2 Stimuli

Stimuli were produced using a custom-built device generating co-located sound and light signals (see [14]). Participants sat in a dimly lit, sound attenuated room with their chins on a chinrest, approximately 60 cm from the device. Audio signals were broadband noise where the peak intensity was 60 dB SPL. Visual signals were generated by a 7×5 red LED array, with aluminance of 41 cd/m^2^. The average signal duration, across trials, was 500 ms, with a 5 ms onset/offset.

**Figure 1:**
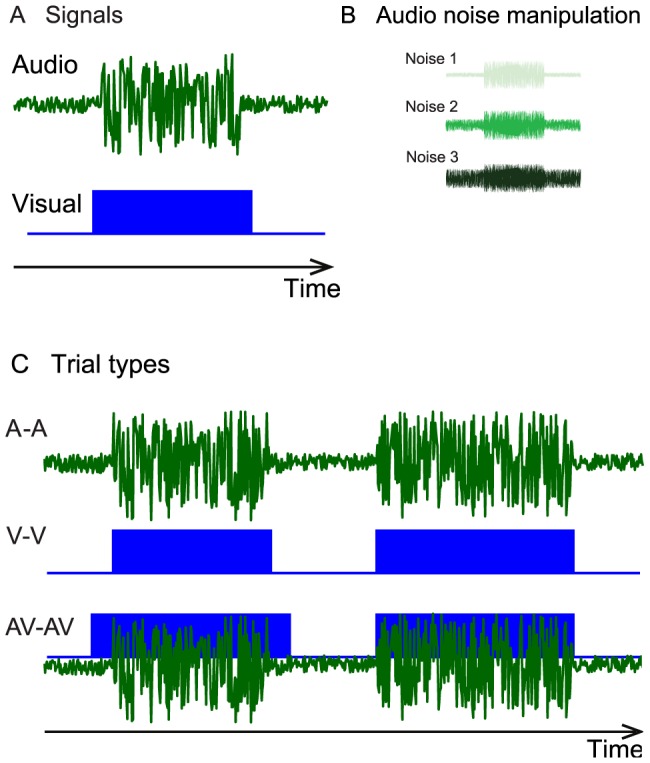
Experiment setup and design. (A) Audio and visual signals defining a filled audiovisual interval. (B) Signal-to-noise manipulation of the audio signal. (C) The three possible trial types: audio, visual, and audiovisual intervals. In each trial one standard and one comparison interval are presented in random order (in the depicted case Interval 1 is the standard as it contains a discrepancy between the audio and visual signals).

In order to alter the reliability of the audio signals we embedded the noise burst (signal) in continuous background noise and manipulated the intensity of this background noise (0.1, 0.6, and 1.2 times signal level, see Figure 1B). The audio background noise was presented throughout the trial randomly spanning between 200 and 450 ms before and after stimulus presentation. No noise was added to the visual signal.

### 2.3 Procedure

We used a two-interval, forced-choice procedure. Each trial consisted of the sequential presentation of two intervals both defined either by audio, visual, or audiovisual signals. Participants indicated which interval lasted perceptually longer (Figure 1c). They received no feedback concerning their response. The inter-stimulus interval (ISI) varied randomly between 500 and 900 ms. The duration of the standard stimulus interval in unisensory trials (i.e. audition alone or vision alone) could be one of three durations: 450 ms, 500 ms or 550 ms; and for the comparison interval, the duration could be ±5, 10, 20, or 40% of the standard interval in that trial, varied according to the method of constant stimuli. The order of standard and comparison was randomized.

In the audiovisual trials, participants compared two intervals where visual and audio signals (with one of three noise levels) were present in both intervals. In these trials the procedure was identical to that implemented in the unisensory trials, only the duration of the audio and visual stimuli of the standard interval contained a small conflict and thus could be different by Δ = 0, −50, +50 ms. The signals were aligned at the temporal midpoint such that the discrepancy was distributed equally at either end of the stimulus. The conflict was introduced in order to measure how each sensory input was updated during integration and to verify if the conflict was resolved according to the unisensosry signal weights. As such the conflicts were implemented to test the weighting predictions of the MLE model. Trials with standard intervals having different discrepancies were presented interleaved in random order.

Each comparison was repeated 16 times. Responses were fit with a cumulative Gaussian from which the points of subjective equality (PSE) and the just noticeable difference (JND) were obtained. The PSE corresponded to the duration at which the proportion of responses ‘comparison appears longer' reached the 0.50 level – thus comparison and standard interval were perceived as equally long. JND was derived by taking the difference in duration between standard and comparison signals necessary to increase discrimination performance from 0.50 to 0.84. The Weber Fraction (WF) was calculated for each condition such that WF = JND/D_S_ where D_S_ is the duration of the standard interval. In this way it was possible to collapse across the three standard durations we tested. For audiovisual stimuli, we took the mean between D_SV_ and D_SA_ as a measure of D_S_. Thus, the WF indicated the inverse of the precision of the duration judgment in the discrimination process. Trials with unisensory and audiovisual stimuli were interleaved throughout the experiment. From the unisensory trials we derived predictions for optimal performance in the multisensory condition according to the MLE Equations (Equations 1–3). This allows us to compare the empirical performance on audiovisual trials with the one predicted from the performance obtained with unisensory stimuli. In the following we will first describe the unisensory results followed by a comparison between the MLE model predictions and empirical findings for the audiovisual trials.

## Results

**Figure 2:**
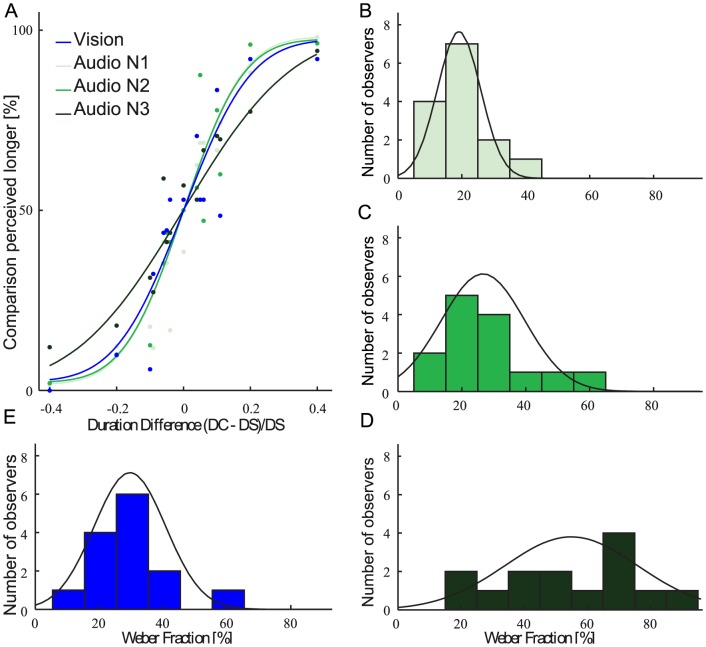
Weber fraction data as a function of noise level. (A) Example participant MDJ's unisensory psychometric functions for the three audio noise levels and for vision. (B) Distribution of Weber fraction values across the 8 participants for the visual condition. (C–E) Distribution of Weber fraction values for the three auditory noise conditions.

Figure 2A shows unisensory visual and auditory WF data for participant MDJ, a representative participant. Audio noise was added to the auditory signal (low, medium, or high noise levels, see Figure 1B) thus modulating the signal-to-noise ratio, but the visual condition was always noise free. For audio stimuli, duration discrimination thresholds increase with increasing noise levels. Visual estimates are approximately as precise as the auditory estimate in the middle noise condition (Figure 2A). Unisensory WF data for all participants are presented in the histograms of Figure 2B–E. The mean auditory WF goes from 19% to 54% as the noise level of the audio stimulus increases. A one-way Repeated Measures (RM) ANOVA comparing the WF for the three different noise levels revealed a significant effect of noise level on WF, F(2,7) = 35, *p*<0.001, η = 0.83. The average visual WF is 29%.

**Figure 3:**
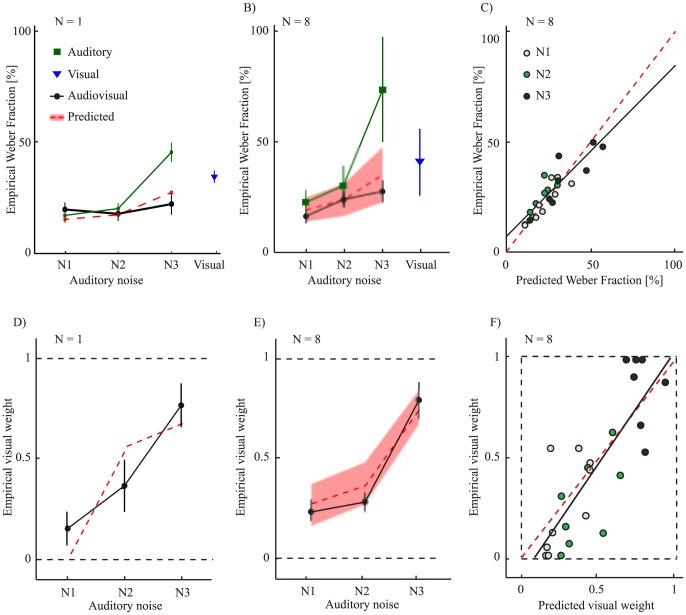
Weber fraction and pse data for the different conditions tested. (A) Example participant MDJ Weber Fraction values for unisensory and, multisensory conditions and MLE predictions. Error bars correspond to the CI from the fitting procedure. (B) Mean unisensory, multisensory, and MLE predicted WF values across participants. Unisensory WF data is obtained from the distributions represented in Figure 2B–E. Predicted values are instead obtained from Equation 7. (C) Relation between empirical and predicted Weber fraction values across participants. For optimal integration, the mapping between observed and predicted should be a 1-to-1 relationship. The line of best fit is consistent with such a mapping. (D) Example participant MDJ's values of PSE_AV_ in multisensory conflict conditions expressed in terms of visual weight. Error bars correspond to the CI from the fitting procedure. MLE predictions indicate that as the noise in the audio signal increases the visual weight should increases correspondingly. (E) Average values of visual weight in multisensory conflict conditions. (F) Individual visual weights showing the correlation between empirical values and predictions for the three noise conditions. The regression line shows the mapping between the predicted and observed weights.

Individual WFs for the unisensory inputs are used to predict performance under multisensory conditions according to the MLE model. For this, Equations 1–3can be applied to the integration of redundant duration information, indicating that perceived duration of audiovisual stimuli D_AV_ should be a weighted average of the audio and visual components such that:
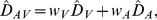
(4)where the weights of the unisensory estimates of duration are calculated using the individual WFs according to
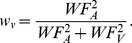
(5)This weight leads to a maximal decrease in uncertainty for multisensory estimates of duration where the Weber fraction in the multisensory conditions can be calculated from the unisensory ones
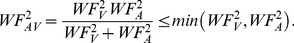
(6)Such predicted reduction of WF in the multisensory condition is verified for participant MDJ in Figure 3A and in Figure 3B-Cacross participants. A 3 (noise level) times 2 (empirical vs. predicted) RM 2-way ANOVA on the WF values does not indicate significant deviations from predictions for empirical values alone (estimate type: empirical vs. predicted: F(1,7) = 1.6 *p* = 0.24, η = 0.28) nor in conjunction with the noise level (estimate type and noise level: F(2,14) = 0.5 *p* = 0.61, η = .15). The natural interpretation of this would be that there is no difference between the empirical and predicted WFs. However finding no significant difference between the predicted and empirical values is not necessarily evidence that they come from the same population. In order to verify this we used the Bayes Factor (BF; see [15]) and quantified the probability that the null hypothesis (no difference between MLE predicted values and multisensory estimates) described the relationship between the two variables. The BF of 3.58 suggests that there is support for the null and therefore that performance in the multisensory condition is well predicted by MLE. Such coherence is also present at the individual level as evidenced by the similarity between predicted and audiovisual observed WF (Figure 3C). A regression line fitted to the data has a slope of 0.76 (95% C.I. = 0.58–0.94) and an intercept of 0.007 (C.I. = 0.02–0.13) with R^2^ = 0.78 (*p*<0.001) indicating that the MLE model successfully predicts the individual performance improvement due to multisensory integration. Moreover, the MLE model predicts that the combined cue estimate is more precise than the best unisensory WF estimate and such an advantage increases when the reliability of the unisensory estimates is comparable. Although previous studies did not find support for this claim (e.g., [8]), here a paired-sample t-test reveals that the best unisensory WF estimate is significantly higher than the multisensory WF for the intermediate noise level (one-tailed paired-sample t-test, t(7) = 3.7 *p* = 0.007, BF = 0.1175). In the other two noise conditions the difference in reliability between the auditory and the visual duration estimates is substantial, thus the predicted improvement in the multisensory condition is small compared to the best unisensory estimate (t(7) = 1.4 *p* = 0.19, BF = 1.726 and t(7) = 1.9 *p* = 0.10, BF = 0.992, for the lowest and highest noise level conditions respectively). This behavior is as predicted by the model: an extreme weight assigned to a sensory component leads to minimal performance advantage. In sum, the decrease in variance associated with audiovisual duration estimates observed in the intermediate noise condition indicates statistically optimal integration for duration.

According to MLE predictions, as the noise in the audio signal increases, participants should rely more on visual information. This can be seen in Equation 5 as the weight (w_A_) assigned to the audio component decreases. The values of PSE_AV_, representing the perceived duration of audiovisual stimuli containing a temporal conflict, can be used to calculate the empirical weights for each participant and verify this prediction. A comparison between predicted and empirical weights at the different noise levels is shown in Figure 3D for participant MDJ and for the average across participants (Figure 3E). As predicted, with increasing audio noise the estimate of duration appears to rely more and more on visual information (the weight given to the audio component decreases). The correlation between predicted and empirical weights is visible in Figure 3F (R^2^ = 0.65 *p*<0.001), which shows a strong correlation between empirical and observed, given that the regression line does not statistically deviate from the line of equality (slope = 1.1, C.I. = 0.77–1.5, intercept = −0.07, C.I. = −0.02–0.10).

Taken together, the results for our variance estimate (WF) and weighting behavior indicate that participants optimally integrate the audio and visual components to obtain a single estimate of multisensory duration.

## Discussion

**Figure 4:**
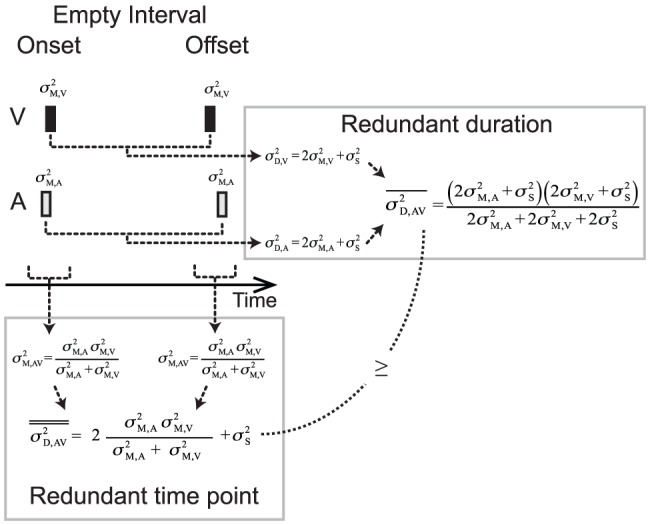
Depiction of a single audiovisual empty interval (i.e., as used by [8]). Short audio and visual onset and offset markers delineate the interval whose duration is to be estimated. If participants integrate redundant unisensory estimates of the interval duration, this would lead to the prediction given in the box titled “redundant duration”. However, it is also possible participants integrate the unisensory estimate of time for the onset and offset markers giving rise to the box titled “redundant time point”. In this second case, the audio and visual markers are first integrated and only at a later stage the duration estimate is made on the integrated markers.

Here we show how the nervous system might obtain an integrated estimate of interval duration for intervals redundantly specified by multisensory signals. We investigated whether such an integrated estimate is statistically optimal, despite the fact that perceived duration of an event can only be obtained post-hoc, i.e., when the sensory information is no longer available. The crucial finding is that redundant audiovisual duration information is integrated in a statistically optimal fashion. That is, multisensory duration estimates are obtained through a weighted average of the unisensory estimates with weights proportional to their reliabilities. This is in contrast with other studies that claimed suboptimal integration of multisensory temporal estimates, particularly for duration ([8]; [9]; [11]). Why this conflict? To gain insight into why, let us consider what information is available for estimating duration in the different studies. All other studies that looked at multisensory integration in the time domain have used short onset and offset markers ([8]; [9]; [16]; [11]). Duration was therefore defined by an “empty interval” between those markers (cf.[9]). Given the empty interval stimulus, multisensory duration estimates could be obtained in one of two ways as suggested in the introduction of this paper (cf. Figure 4). Here we formalize these two options:

1. The nervous system could either obtain an estimate of duration for the audio and visual channels independently from the auditory and visual empty interval, respectively. The two redundant estimates of duration could then be integrated into a unified estimate of multisensory duration. We named this case “redundant duration” to distinguish it from the alternative way integration could have been achieved, which we termed “redundant time point”. For “redundant duration” multisensory duration estimation performance should be predictable from the unisensory duration estimates following Equations 4–6.
2. Alternatively, for the short multisensory onset and offset markers defining the empty interval, it could be that the multisensory signals demarcating the markers are first integrated into a multisensory estimate of the time points marking the beginning and the end of the interval. Duration judgments could then be made based on these integrated onset and offset markers and not on integrating two redundant duration estimates. In such a case, predictions cannot be made using Equations 4–6, rather they follow a different scheme as described below.

Let us compare the performance that can be achieved in duration judgments with the two proposed models that involve either integration of duration information, or of time points provided by the markers. In either case, the precision of a duration estimate is limited by two noise sources, one due to the signals marking start and end of the interval and one source due to storage of the temporal information from onset until offset. The latter component represents a noise source in the duration estimate that is coming from memory and which makes longer durations more difficult to discriminate than short ones. The variance 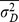 of a duration judgment can be then expressed as
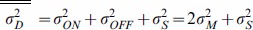
(7)where it is assumed that the variances associated with the onset and offset are equal (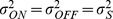).

1) If integration occurred according to the “redundant duration” model the variability of the integrated estimate 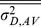
can be expressed as a function of the variability of the unisensory estimates 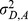 and 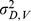 following Equation6: 
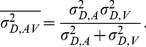
(8)Substituting the unisensory variances 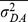 and 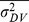
with Equation7 (and assuming that 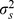 is equal in the two modalities) expands to:
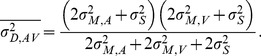
(9)The same substitution can be done for Equation5 to obtain the weight assigned to the unisensory estimates as a function of markers and storage noise 
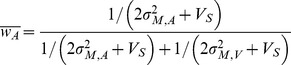
(10)The single line superscripts in Equations 8–10 indicate estimates according to the “redundant duration” model to distinguish them from the “redundant time point” model.

2) If instead integration occurred according to the “redundant time point” model, the audio and visual markers are first integrated and duration is then estimated from their difference. Thus, the variability of the onset and offset markers becomes 
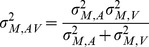
(11)and the weight given to the two markers is instead a function of the marker's reliabilities according to 
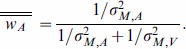
(12)The two-line superscript indicates estimates according to the “redundant time point” model. This leads to a variability of the duration estimate of audiovisual conditions that is expressed by
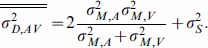
(13)


If we now compare the two models, it is easy to demonstrate that if the storage of temporal information does not cause a decrease in performance for multisensory duration judgments (i.e., if 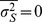), then there is no difference in the two methods of integration both in terms of variability (Equation 9 is equal to Equation 13) and weighting of the audio and visual components (Equation 10 is equal to Equation 12). However, if 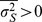 then the variability of duration judgments based on integrated markers described by Equation13 is necessarily larger than the one obtained through integration of redundant duration information expressed by Equation 9. This means that with this additional term the integration of time point information leads to less reliable duration estimate than the integration of duration information (Figure 4). The reason for this imbalance is that when the estimate of time points is treated as redundant, the duration estimate is derived from an integrated time point thus the noise term associated with the memory storage component 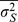 appears as an additional factor in variance (Equation 13), but it is not considered in the weighting (Equation 12) leading to suboptimal integration. In fact, the variances determined for the time estimate of the makers may differ from the variances of the duration estimate because of the additional memory storage term. That is, the variance of the duration estimate is determined by more than just the variance of the markers (there must be an additional noise source in the duration estimate which decreases the absolute precision of duration estimates as duration increases). However, the precision in determining a point in time for the onset and offset alone should be largely independent of the duration that passes between those markers. As a consequence, if the variance associated with the two modalities is not equal, the weight assigned to the most reliable component (in the time domain, this is normally the auditory modality) is higher for the “redundant time point” model than for the “redundant duration” model. Therefore, integrating information from markers rather than for duration would be associated with overweight of the auditory modality and for an associated higher variance for the overall duration judgment. This could explain the apparent contradiction between our result showing optimal integration and the ones of Burr et al. [8] that shows overweighting of auditory information. It must also be noted that the current study employs intervals that are defined by the continuous presence of stimuli. Integration at the level of marker time points with this kind of stimuli is difficult to conceive, and we argue that this is the reason why we correctly predicted optimal performance whereas others employing empty intervals did not.

Duration estimation differs from all the other dimensions investigated for redundant cue combination for which optimal integration has been found, in that it is a post-hoc estimate. That is, the perceptual judgment cannot be made while the sensory information is still available. Duration can only be judged after the event has terminated. Being optimal in integrating multisensory event duration therefore means that the integration mechanism operates on the stored representation of event duration and its associated variance.

Recent findings highlight the importance of models of duration reproduction (see [17]; [18]) where it has been suggested that both humans [18] and rats [19] are able to learn distributions of duration and, in a ready-set-go task, reproduce the go duration optimally. Jazayeri and Shadlen [18] showed that prior information about the experienced duration distribution is used in reproducing temporal intervals. They asked participants to reproduce time intervals that were sampled from different underlying distributions (including sub-second intervals). The resulting reproductions of target intervals were observed to regress to the mean according to a Bayesian model that included a cost function and a prior distribution of the range of duration presented. These findings have been more recently confirmed with different populations (i.e., showing a lesser influence of the prior for musicians, [17]) and they are in line with the current results as they demonstrate that both duration estimation and reproduction of a temporal interval lead to statistical optimality according to Bayesian inference models that include a prior distribution (i.e., [18]; [17]) or that rely solely on likelihood functions as in this study.

Evidence of optimal integration for perceived duration is further intriguing as it provides insight into the current debate of how neural timekeeping mechanisms are implemented in the nervous system. Duration estimation has been used to understand how temporal information is coded and processed in humans and animals a like [20]; [21]; [1]). The prevailing view is that temporal judgments rely on a centralized internal clock or pacemaker feeding into an accumulator ([22]; [23]). More recent models, however, consider distributed timing networks, with different mechanisms timing different interval lengths ([2]; [1]; [24]; [25]). Heron et al. [25] used an adaptation procedure and suggested that there are multiple channels tuned to specific bandwidths of duration. Such channel models are well established albeit contentious for properties such as spatial frequency ([26]; [27]; [28]). Since MLE successfully describes the increase in performance, this indicates that redundant multisensory components are statistically independent. If this was not the case, lower performance should have been obtained [29]. The current findings are thus incompatible with the notion that duration estimation may be obtained through a unique or partially dependent duration mechanism and is thus in agreement with independent channel models(i.e., [25]; [30]).

Estimating elapsed time is a critical ability for many other perceptual and cognitive functions (e.g., [31]). It is known that perceived duration is modulated by the emotional state of the observer [32], by voluntary actions [33] or by making a saccade ([34]; [35]; [36]). We demonstrate that perceived duration of audiovisual events depends upon the reliability of the information the nervous system is sampling. Others have shown that perceived time is altered by the magnitude of the signal, which could affect its reliability, though this was not measured [37]. We would suggest that it therefore highlights the plasticity of a candidate timing mechanism. However, our results suggest that a pace-maker accumulator implementation that would involve a different clock for each modality is not the most parsimonious description. When experiencing elapsed time in the ms-sec range the perceived duration appears to be modulated by signal statistics more than modality specific constraints.

### Conclusion

The nervous system can integrate redundant information about the temporal extent of a multisensory event so as to reduce the uncertainty in its perceptual estimate of duration. The performance is close to optimal when information about unisensory duration is integrated, in contrast to the case when the time points defining the interval are integrated. For duration estimates the nervous system integrates redundant information in a manner similar to spatial estimates, despite the fact that the information is no longer available when the estimate is made. It makes sense that estimating properties of the environment would involve a variance minimization process where possible. So, next time you go to a concert, make sure you watch and listen!
