# Correction: The Duration of Uncertain Times: Audiovisual Information about Intervals Is Integrated in a Statistically Optimal Fashion

**DOI:** 10.1371/journal.pone.0096134

**Published:** 2014-04-18

**Authors:** The PLOS ONE Staff

**Notice of Republication**

This article was republished April 3, 2014, to correct numerous spacing errors throughout the text that were introduced during the typesetting process. Please download this article again to view the correct version. The originally published, uncorrected article and the republished, corrected article are provided here for reference. The publisher apologizes for this error.

## Supporting Information

File S1 Originally published, uncorrected article.
(PDF)

File S2 Republished, corrected article.
(PDF)
